# F43. POTENTIATION OF INHIBITORY NEUROTRANSMISSION IN THE TREATMENT OF RECENT-ONSET SCHIZOPHRENIA BY MODIFICATION OF DEVELOPMENTAL PRUNING OF PREFRONTAL CIRCUITRY

**DOI:** 10.1093/schbul/sby017.574

**Published:** 2018-04-01

**Authors:** Erin Burke, Joanne Wojcik, Larry J Seidman, Alan Green, Tsung-Ung Wilson Woo

**Affiliations:** 1 Arbour Hospital; 2 Beth Israel Deaconess Medical Center, Harvard Medical School; 3 Dartmouth College; 4 Harvard Medical School/McLean Hospital

## Abstract

**Background:**

The overt symptoms and deficits of schizophrenia (SZ) typically emerge during late adolescence and early adulthood, followed by a period of post-onset functional deterioration. This peri-onset period temporally coincides with the final maturation of the prefrontal cortex (PFC), which is characterized by a process of extensive pruning of synaptic connectivities. Developmental maturation of inhibitory neurotransmission may play a key role in regulating the onset and duration of peri-adolescent synaptic pruning. We hypothesize that a deficit in the developmental increase in inhibitory neurotransmission may disturb the PFC synaptic pruning process and hence contribute to the onset and the functional deterioration that is characteristic of the early course of SZ. Enhancement of inhibitory neurotransmission may therefore restore the integrity of PFC neural circuitry, which may then lead to lasting improvements in cognitive deficits and clinical symptoms.

**Methods:**

Here, we report preliminary data on the possible efficacy of tiagabine (Gabitril), which is a selective uptake inhibitor of the GABA (gamma-aminobutyric acid) transporter GAT-1, in the treatment of recent-onset schizophrenia. Subjects were randomized to receive either tiagabine or placebo added on to their antipsychotic regimen.

**Results:**

Our data suggest that treatment with tiagabine during the early course of the illness can modulate PFC activation, as demonstrated by functional magnetic resonance imaging during working memory, and improve negative symptoms.

**Discussion:**

Taken together, the proposed treatment strategy represents an effort to actively translate preclinical findings in SZ research into clinically testable hypotheses. This kind of translational approach, we believe, will ultimately lead to breakthrough in the treatment and possible prevention of SZ.

